# Hyperspectral imaging and deep learning for parasite detection in white fish under industrial conditions

**DOI:** 10.1038/s41598-024-76808-w

**Published:** 2024-11-09

**Authors:** Shaheen Syed, Samuel Ortega, Kathryn E. Anderssen, Heidi A. Nilsen, Karsten Heia

**Affiliations:** 1 Department of Seafood Industry, Nofima AS, P.O. Box 6122, 9291 Tromsö, Norway; 2 Department of Computer Science, UiT, The Arctic University of Norway, Hansine Hansens Veg 18, 9009 Tromsö, Norway

**Keywords:** Applied physics, Imaging techniques

## Abstract

Parasites in fish muscle present a significant problem for the seafood industry in terms of both quality and health and safety, but the low contrast between parasites and fish tissue makes them exceedingly difficult to detect. The traditional method to identify nematodes requires removing fillets from the production line for manual inspection on candling tables. This technique is slow, labor intensive and typically only finds about half the parasites present. The seafood industry has struggled for decades to develop a method that can improve the detection rate while being performed in a rapid, non-invasive manner. In this study, a newly developed solution uses deep neural networks to simultaneously analyze the spatial and spectral information of hyperspectral imaging data. The resulting technology can be directly integrated into existing industrial processing lines to rapidly identify nematodes at detection rates (73%) better than conventional manual inspection (50%).

**Supplementary Information:**

The online version contains supplementary material available at 10.1038/s41598-024-76808-w.

## Introduction

Parasites in fish are a significant problem for seafood producers world-wide, presenting both quality and health concerns. For Atlantic Cod (*Gadus morhua*), there are two main species of nematodes that are typically found to infest the fish^1^. The first is the *Pseuodoterranova decipiens*, also known as the seal worm. These can grow to be up to 1 cm in diameter when curled and are often dark red or brown in color, particularly the larger specimens. *P. decipiens* nematodes can be found throughout the muscle tissue, including the loin and tail of the cod^2^. The second type of common nematode in cod is the *Anisakis simplex*, sometimes referred to as the herring worm. These nematodes tend to be smaller (up to 3-4 mm in diameter when curled), white or yellowish in color, and restricted to the belly area of the cod fillet^2^. Because of the nematode life cycle, infection tends to be worse for fish that reside near the coastal regions^3,4^.

Consumption of fish containing nematodes can lead to different types of health problems. If fish infected with nematodes is consumed raw or undercooked, this can lead to human infection by the nematode larva^5,6^. The larva burrow into the gastrointestinal walls, where they encapsulate and eventually die, leading to a painful syndrome referred to as intestinal anisakiasis^7^. In rare cases, individuals may have a potentially lethal allergic reaction to the Anisakis protein in cooked fish^8–11^.

Because of the strong disgust factor in finding nematodes in fish, the tolerance for parasites by seafood producers and purchasers is extremely low as it creates negative economic consequences from product rejection, decreased marketability of fish, and loss of consumer trust in seafood products^12–16^. These in turn can lead to both economic and job losses within the fishing industry. Therefore, seafood producers typically screen cod for nematode infection. The current state-of-the-art method for screening for parasites involves manual examination using an illuminated table, also known as a candling table. After the cod has been filleted, workers take the fillets from the conveyor belt, place them on a candling table, and quickly examine for parasites. The workers then trim away any nematodes that are discovered. The drawback of this technique is that it is time consuming and resource intensive, often accounting for up to 50% of the production costs of fillets^17^. Additionally, the method is subjective and there is often a significant percentage of nematodes that are not detected. Research indicates that only around 50% of nematodes are found under industrial screening conditions^18,19^. If the skin is still on the fillet, this drops to a 25% detection rate^2^.

Therefore, the development of an automated technology that could be installed along a conveyor belt to screen seafood for nematodes would have significant benefits in terms of improved product safety, quality, and lowered labor costs. However, detection of nematodes in tissue is a non-trivial problem. The low contrast between nematodes and the surrounding tissue makes them exceedingly difficult to detect. Numerous technologies^20,21^ have been explored without success, such as ultrasound^18,22^, UV illumination^14,23,24^, conductivity^22^, electromagnetism^25,26^, and magnetometry^27^. Despite decades of research, even the most promising techniques have only produced marginal results. Magnetic resonance imaging, although promising in detecting nematodes, is too slow and expensive to use in an industrial setting^28^. Investigations involving x-ray techniques have struggled with low or non-existent contrast between the nematodes and surrounding tissue^29^. The most promising technology to date has been hyperspectral imaging. A normal camera measures three wavelengths in each pixel: one in the red, blue, and green regions of the visible light spectrum. A hyperspectral camera is similar but measures numerous wavelengths of light in each pixel, hence highly detailed information in both the spectral and spatial dimension. The spectrum in each pixel gives information on the chemical and physical structure of the sample in that location.

Hyperspectral imaging has long been investigated as a possible detection method of nematodes in seafood^30–32^. Initially, the technology was too slow and cumbersome to be used outside of a research setting. However, with technological advancements, hyperspectral equipment and the surrounding analysis have become cheaper and faster, such that the technology is now viable for screening seafood in an industrial setting. The hyperspectral imaging system used for this study, the Maritech Eye, is now commercially available to screen for several other quality defects in seafood^33^.

An early study using interactance hyperspectral imaging showed a good detection rate of nematodes compared to visible inspection^34^ but was not able to perform at the speed needed for industrial requirements. A follow-up study^29^ some years later with improvements to the equipment was able to measure rapidly enough for commercial application but produced worse detection rates. Xu et al. performed detection of nematodes in grass carp shashimi samples under laboratory conditions^35^. That work found that the difference in the spectra between nematodes and the fish flesh is very minor, highlighting the challenge in detecting nematodes on a spectral solution alone. A limitation of previous hyperspectral studies is that only the visible or spectral information could be used at one time given the available analysis tools. The advent of deep learning has opened up the ability to simultaneously use both spatial and spectral information in developing detection algorithms. Recent research has been carried out using deep learning techniques to integrate both the spatial and the spectral information from multispectral and hyperspectral imaging^36,37^. The current study uses hyperspectral images of cod fillets acquired under industrial conditions to create deep learning neural network models to detect parasites in fish tissue in real time.

## Results

To evaluate the overall detection performance of nematodes by the models, we use the quality metrics of precision, recall, and F1 score. Precision is the ratio between the actual number of successfully detected nematodes and the total number of nematodes that the model flagged. High precision indicates an accurate detection of positive cases with a low number of false positives. The recall metric calculates the ratio of true positives (nematodes effectively identified by the model) against all positive cases in the dataset (all nematodes existing in the dataset). In our application, a high recall value points to an accurate nematode identification, with a reduced rate of false negatives. Finally, the F1 score is the harmonic mean of precision and recall and measures the overall quality of prediction. Unlike the overall accuracy, precision, recall, and F1 are not sensitive to unbalanced datasets.

These evaluation metrics rely on two different thresholds. The model outputs a value between 0 and 1 for each pixel in the hyperspectral image, indicating the probability of that pixel belonging to a nematode. The model threshold determines the probability level required to assign a pixel to the class nematode. The intersection-of-union (IOU) metric measures the overlap between the pixels identified as nematode by the neural network model and the pixels marked as nematode by manual annotation of the images. The IOU threshold determines how much overlap is necessary to be considered a correct detection. The higher the IOU threshold, the higher the required overlap between the predictions and the annotated pixels to be considered a positive detection. The IOU threshold impacts the evaluation metrics as higher IOU thresholds require a higher match between the bounding boxes.

Table 1 shows the evaluation metrics for a model threshold of 0.5, which means that only pixels with more than 0.5 probability of belonging to a nematode are assigned to that class. The performance metrics for IOU threshold values ranging from 0.1 to 0.9 are presented. Higher IOU thresholds force a more precise alignment between the annotated data and the neural network model predictions, requiring a higher match in the position of the predicted nematodes and the actual position.

**Table 1:** Nematode detection performance depending on the IOU.

	Train			Validation			Test		
IOU	Precision	Recall	F1	Precision	Recall	F1	Precision	Recall	F1
0.1	0.892 (± 0.004)	0.757 (± 0.005)	0.819 (± 0.005)	0.889 (± 0.022)	0.735 (± 0.031)	0.804 (± 0.028)	0.902 (± 0.022)	0.775 (± 0.031)	0.833 (± 0.027)
0.2	0.892 (± 0.004)	0.757 (± 0.005)	0.819 (± 0.005)	0.889 (± 0.022)	0.735 (± 0.031)	0.804 (± 0.028)	0.9 (± 0.022)	0.761 (± 0.031)	0.824 (± 0.028)
0.3	0.89 (± 0.004)	0.743 (± 0.005)	0.81 (± 0.005)	0.889 (± 0.022)	0.735 (± 0.031)	0.804 (± 0.028)	0.9 (± 0.022)	0.761 (± 0.031)	0.824 (± 0.028)
0.4	0.886 (± 0.004)	0.711 (± 0.006)	0.789 (± 0.005)	0.882 (± 0.022)	0.684 (± 0.032)	0.77 (± 0.029)	0.898 (± 0.022)	0.746 (± 0.032)	0.815 (± 0.029)
0.5	**0.878 (± 0.004)**	**0.659 (± 0.006)**	**0.753 (± 0.005)**	**0.873 (± 0.023)**	**0.633 (± 0.034)**	**0.734 (± 0.031)**	**0.897 (± 0.022)**	**0.732 (± 0.033)**	**0.806 (± 0.029)**
0.6	0.863 (± 0.004)	0.578 (± 0.006)	0.693 (± 0.006)	0.85 (± 0.025)	0.52 (± 0.035)	0.646 (± 0.033)	0.878 (± 0.024)	0.606 (± 0.036)	0.717 (± 0.033)
0.7	0.817 (± 0.005)	0.411 (± 0.006)	0.547 (± 0.006)	0.809 (± 0.027)	0.388 (± 0.034)	0.524 (± 0.035)	0.857 (± 0.026)	0.507 (± 0.037)	0.637 (± 0.035)
0.8	0.694 (± 0.006)	0.208 (± 0.005)	0.32 (± 0.006)	0.71 (± 0.032)	0.224 (± 0.029)	0.341 (± 0.033)	0.769 (± 0.031)	0.282 (± 0.033)	0.412 (± 0.036)
0.9	0.443 (± 0.006)	0.073 (± 0.003)	0.125 (± 0.004)	0.55 (± 0.035)	0.112 (± 0.022)	0.186 (± 0.027)	0.684 (± 0.034)	0.183 (± 0.029)	0.289 (± 0.033)

“Significant values are in [bold]”

The evaluation metrics are similar for the training, validation, and test data, suggesting no overfitting. The results achieved on the test set are slightly more accurate than those for the training and validation sets. This can be caused by the random partition of the data, where the training and validation datasets could contain more challenging instances than the test set. The results also indicate that the model is characterized by high precision and moderate recall, which means that the number of false positives is low compared to the number of false negatives.

For the test set, the results for an IOU threshold ranging from 0.1 to 0.3 are similar, with values for precision, recall, and F1 score higher than 0.9, 0.76, and 0.82, respectively. The high precision indicates the model is able to identify nematodes with a high number of true positives and a low number of false positives. The value for recall suggests that the neural network model is able to detect 76% of the nematodes, while 24% of the nematodes are not detected (false negatives). Finally, the F1 score provides an average between precision and recall. For an IOU threshold of 0.5, the precision, recall, and F1 score for the test set are 0.89, 0.73, and 0.80, respectively. This means slightly worsened results but still very competitive. However, the evaluation metrics are negatively affected when the IOU threshold exceeds 0.5. The number of nematodes detected for IOU thresholds of 0.6 and 0.7 decreased to 60% and 50%, respectively, and higher IOU thresholds downgraded the nematode detection rate to less than 30%. The interpretation of the results of varying the IOU threshold suggests the model is able to detect nematodes, but there arise some differences in the exact pixels predicted when compared to the manual annotation. At 0.5 IOU, which is selection to be considered the optimal threshold for the developed model, the detection rate (73%) was significantly better than the industrial rate (50%).

**Fig. 1:**
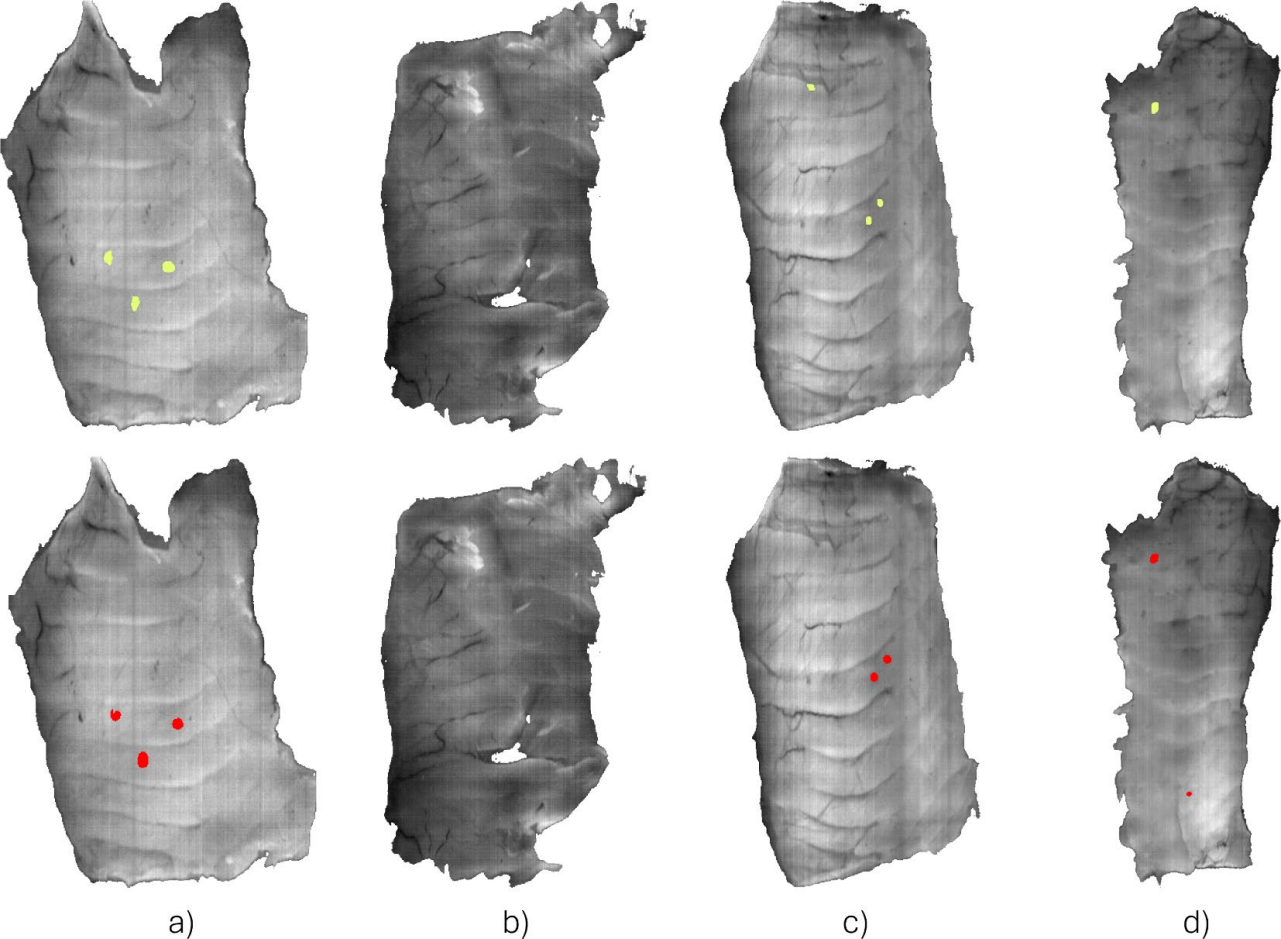
Example of different results for the automatic detection of nematodes with hyperspectral imaging and the proposed processing approach. (**a**, **b**) Successful detection of nematodes. (**b**) Example of a false negative. (**c**) Example of a false positive.

Figure 1 shows the graphical results of the nematode detection algorithm in examples from the test set. The images on the top row represent the position of the nematodes according to the manual annotations (yellow). The bottom row shows the predictions by the neural network model, where the segmentation of the nematodes is shown in red. Figure 1.a shows an example of a perfect detection, where all three nematodes in the samples were detected. Figure 1.b shows another successful outcome of the model, but this time in a nematode-free sample. Figure 1.c is an example of a non-detection of a nematode by the model, i.e., a false negative. However, the remaining two nematodes were successfully detected. Finally, Fig. 1.d shows an example of a false positive. In this case, the only nematode present in the sample was correctly detected, but the model predicted a nematode in an area free of parasites.

## Discussion

This research study demonstrates the capability of hyperspectral imaging and deep learning techniques to automatically identify nematodes embedded in cod muscle under industrial conditions at detection rates better than manual inspection. This ability is attributed to two main reasons. First, the high-powered interactance illumination setup used by the hyperspectral equipment makes it possible to have information on not only the surface of the fish muscle, but also information about deeper layers, enabling detection of nematodes embedded within the fish flesh. Such strong levels of illumination cannot be used for visual inspection as they overwhelm the human eye. Also, because the sample is illuminated from the same side as the detection camera, the thickness of the samples is less important and will not be negatively impacted by the presence of skin. In contrast, the conventional screening method using a candling table relies on light passing through the samples. Therefore, for thicker samples or samples with the skin on, the amount of light able to penetrate the fish tissue is reduced and hinders the ability of trimmers to see parasites.

The second reason lies in the combination of monotony and physical demands inherent in the job of the trimmers. These workers are tasked with meticulously inspecting and removing parasites from seafood products, a tedious process that demands prolonged attention to detail. The monotony of the job is emphasized by the repetitive nature of the task. Inspecting each fillet to ensure it meets quality standards requires an unwavering focus, as missing even a single parasite can lead to rejection of the entire delivery. Moreover, the physically demanding nature of the job adds an extra layer of challenge. The constant repetition of motions, such as trimming and inspecting, can lead to fatigue and strain on the body. Standing for extended periods and performing intricate tasks with precision can take a toll on the trimmers, making it a demanding job that requires both mental and physical resilience. In contrast, unlike people, the algorithms never grow tired or distracted.

These results have important implications for the seafood industry. Currently, every fillet must be manually inspected for nematodes. Using hyperspectral screening, only those with fillets identified with nematodes will need to be passed along for trimming and the annotated hyperspectral images can be used to assist the trimmers in removing the parasites. This will significantly reduce labor costs and the burden on the trimmers. The percentage of fillets infected with nematodes varies greatly depending on location and time of year. For some regions, like the Barents Sea, almost all specimens are infected while the number is much lower for cod from the North Sea. On average, about half of all cod have some level of nematode infection^3^. Automatic detection of nematodes by the seafood industry also opens up the possibility for researchers to collect statistics and assess the prevalence and impact of nematode infestations at scales far beyond what is possible in research studies alone.

Although the initial results of the study are promising, there remain several areas for improvement. The proposed method necessitates a considerable sample size for proper training and evaluation, and thus, expanding the sample set could lead to improved detection rates. However, during the different data collection campaigns, we emphasized the quality of data rather than its volume. Our objective was to gather a considerable number of images, ensuring that the annotations were accurate for effective algorithm training and evaluation. While we acknowledge that the available sample for our study is limited (comprising 289 annotated images and 244 nematodes), it is worth noting that this sample size significantly exceeds the sample sizes of other studies related to nematode detection using hyperspectral imagery^29,34–38^. Additionally, as there is seasonal variation in the hyperspectral response of fish tissue, samplings from multiple times of year should be included in the training set to improve robustness. It is expected precision and recall can be improved using a larger dataset with a larger variation in the number of nematodes, their size, species, and position in the fish muscle. Additionally, detection rate could be improved by scanning at a lower speed, making a trade-off between throughput and identification. The solution presented in this work is limited to samples corresponding to the belly flaps. This is where the majority of nematode infestation occurs, but the region is not as thick as other parts of the fish, such as the loin. An important challenge to improving models lies in the difficulty in providing the ground truth of where nematodes are located in the fillets. Translating where a nematode was found during inspection to its location in a hyperspectral image is non-trivial due to deformation of the tissue during handling. Furthermore, even when fillets are inspected for nematodes by laboriously slicing the fish into thin sections, some nematodes are missed. There have been anecdotal incidents where apparent false positives by the neural network model turned out to be nematodes not detected upon manual evaluation of the fillets. Furthermore, it has been found that some false positives arise from other types of quality defect in the fillet (e.g. a blood spot, piece of black membrane) that can be removed by the trimmers to improve the visual quality of the fillet^29^. To address the issues associated with the subjective ground truth in future experiments, it is possible to utilize other technologies capable of precisely locating nematodes within samples, such as MRI or CT scans. However, the slower processing times of these technologies will restrict the number of samples with a more accurate ground truth that can be analysed.

Additionally, the conditions under which the hyperspectral images are captured (exposure time and the conveyor belt’s speed) determine the pixels’ spatial resolution. In this case, the acquisition conditions allowed to have square pixels, which means that the morphology of the fish samples and the nematodes are kept intact. However, different combinations of the acquisition parameters will produce images showing a morphological deformation, resulting in images stretched in the horizontal or vertical direction. Those deformations will affect the shape of the nematodes and will likely affect the model’s performance since it uses spatial information for the predictions. In this study, all data were gathered from a single factory. The performance of the method should remain consistent as long as the quality of the acquisition procedures and raw materials is maintained. However, variations on the acquisition parameters, especially the speed of the conveyor belt, could distort the shapes of the nematodes, negatively affecting the results. In such cases, it would be necessary to retrain the algorithm with data collected using the specific acquisition parameters of the new facility. Integrating supervised learning with other techniques, such as unsupervised learning, anomaly detection, or more sophisticated dimensionality reduction methods could improve the accuracy of the predictions. However, in our preliminary evaluation of methods for detecting nematodes, we found that unsupervised techniques and anomaly detection methods failed to identify the location of the parasites. Further exploring these techniques could not only improve the performance of supervised learning methods but also assist in digitizing annotations within hyperspectral images, a process that is currently manual and labor-intensive.

In conclusion, we present a working solution based on hyperspectral imaging and deep learning for detecting nematodes under industrial conditions. The data was recorded under real world processing conditions, and the model execution performance to provide results in real-time has also been tested in an industrial environment. The algorithm can be executed at a throughput of 500 frames per second (exposure time of 2 ms). Although there is room for improvement in the detection rate, especially in the recall, this research work proves that the detection of nematodes is possible using hyperspectral imaging and neural networks, which can be used in seafood processing factories to improve the current workflow for nematode inspection and trimming. Already, the seafood industry has begun adoption of the technology. Based on the work described in this paper, a Maritech Eye has recently been installed at a filleting factory in Iceland to perform industrial sorting of cod fillets for nematodes.

## Methods

### Hyperspectral instrumentation

The hyperspectral data were captured using a Maritech Eye (Maritech Systems AS, Molde, Norway), an industrial-grade hyperspectral imaging system. This device is comprised of an interactance illumination system, a hyperspectral camera, and a high-performance processing unit. The interactance illumination can simultaneously measure both the absorption and scattering properties of biological tissues. Unlike the more commonly used diffuse reflectance illumination setup, interactance setups can measure beyond the sample surface to measure properties within. Depth of penetration in interactance hyperspectral imaging is dependent on a variety of factors including light intensity, distance between the light lines and, the optical properties of the sample, such as absorption, scattering, and refractive index. Depth of penetration also varies with the wavelength of light. Absorption dominates at shorter wavelengths, leading to shallow penetration, while scattering becomes more significant at longer wavelengths, allowing light to travel deeper into the tissue. Although to date the relationship between these factors has not been developed theoretically, there have been several experimental studies regarding light penetration in fish tissue^39,40^. Empirically, the depth of penetration is typically in the 1-2 cm range, though in some cases deeper measurement is possible^25^. The Maritech Eye allows the acquisition and processing of hyperspectral images in real time and has been designed as an industrial solution for the seafood industry.

The hyperspectral camera is a HySpex Baldur V-1024N (Norsk Elektro Optikk AS, Oslo, Norway), which is a pushbroom camera covering the VNIR (visual and near-infrared) spectral range (485-960 nm) with a spectral resolution of 5.5 nm (88 spectral bands) and 1024 spatial pixels. The field of view is approximately 300 mm at a working distance of 1000 mm. As a pushbroom camera, each frame contains only the spectral information of a single narrow spatial line. Numerous frames are taken as the target sample moves along a conveyor belt, creating a hyperspectral data cube. The speed of the conveyor belt for this study was set to 130 mm/s, the standard configuration for the fish factory where measurements were taken. With this setup and an exposure time of 2 ms (500 fps), the final pixel size of the measurements was approximately 0.27 × 0.26 mm. The interactance illumination is comprised of two focused halogen light lines (900 W electrical power). These focused lights are separated by approximately 8 mm, and the focusing plane of the hyperspectral camera is located between them^41^.

### Raw material and data labeling

The Maritech Eye instrumentation was used to take hyperspectral images of cod belly flaps, both with and without nematodes. A total of 335 samples were gathered and analyzed from a fish factory in Portugal from three different sampling dates, spanning from December 2020 to June 2022. The fish samples for the study were obtained from commercial fillet production. The samples consisted of belly pieces from Atlantic cod (*Gadus morhua*) caught in Russian, Norwegian, and Icelandic waters. The average length of the belly pieces was 19.4 ± 3.9 cm, with an average width of 10.1 ± 1.8 cm.

**Fig. 2:**
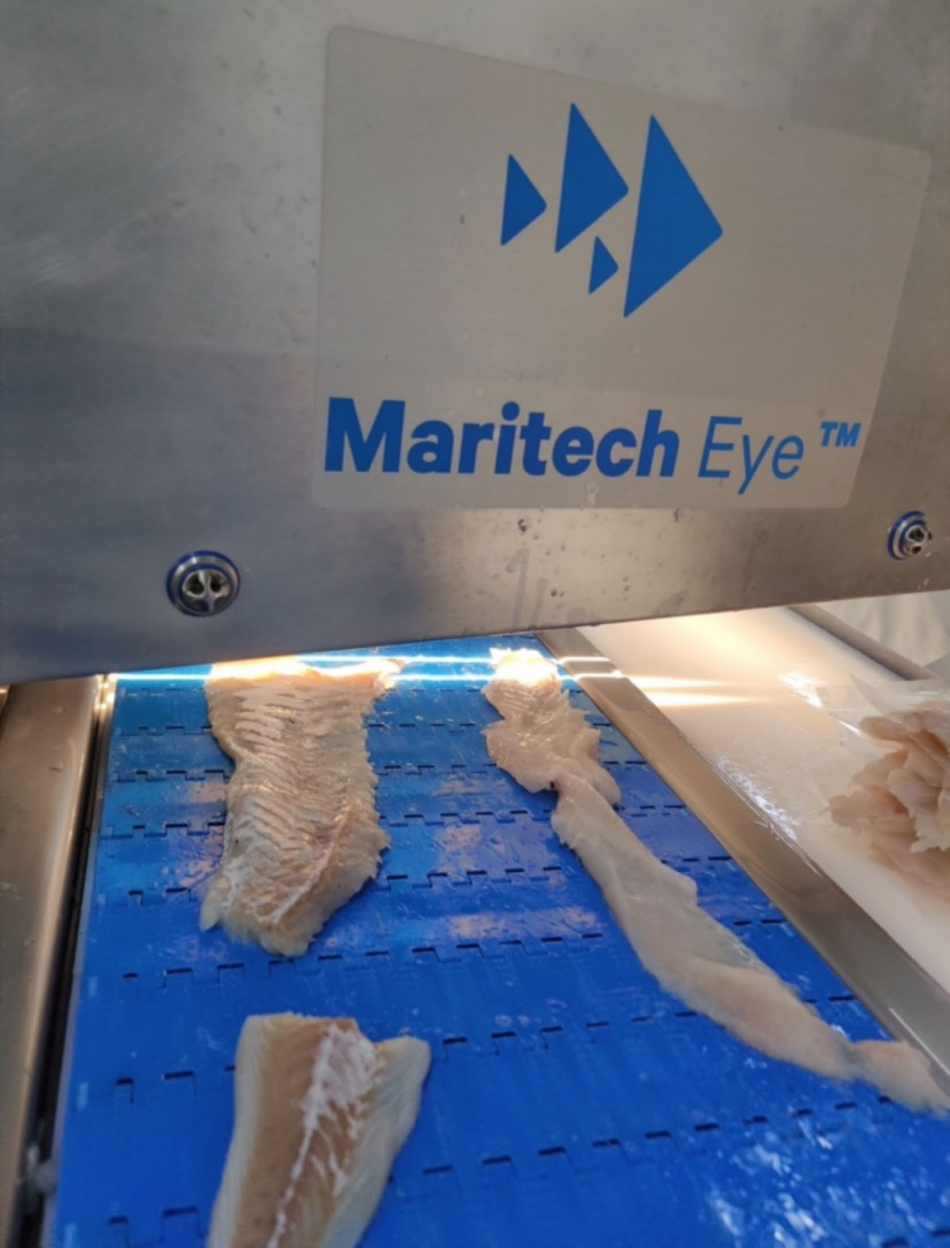
Maritech Eye hyperspectral instrumentation being used in the processing line under industrial conditions.

**Fig. 3:**
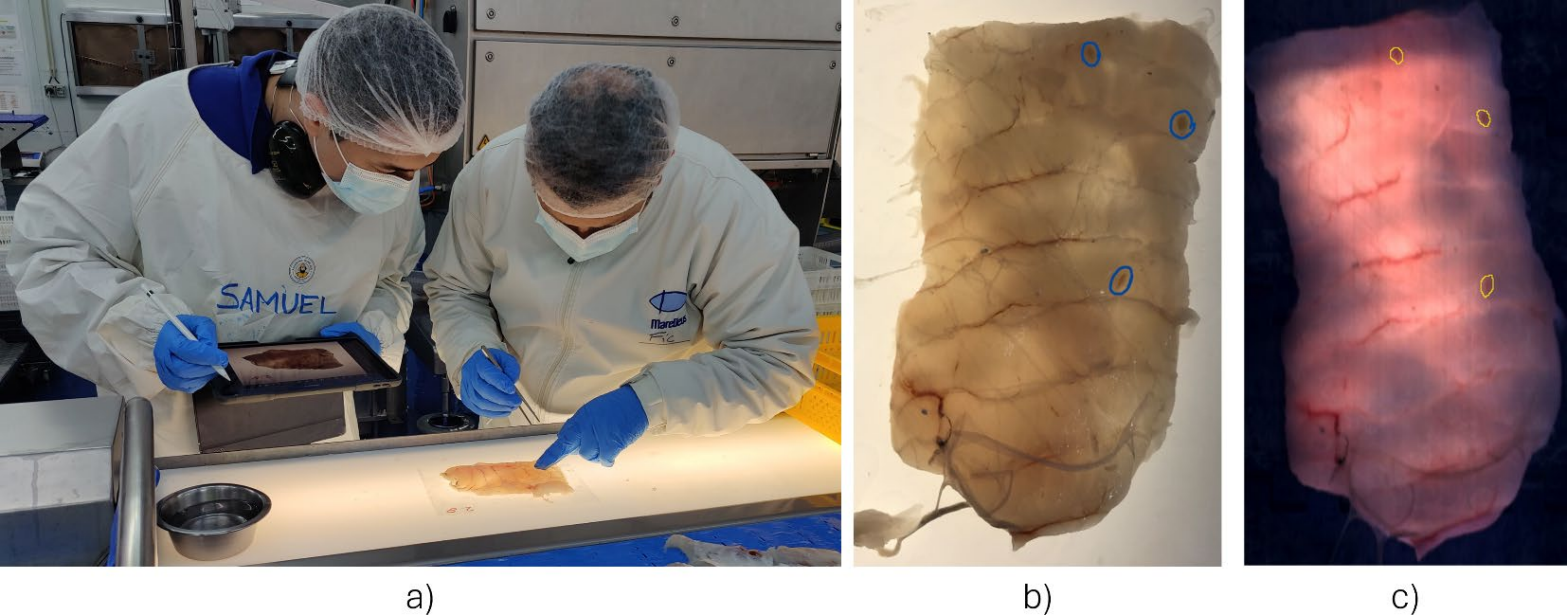
Workflow for the identification and labeling of nematodes. (**a**) Manual examination of the fillets for nematodes using a candling table, and digital annotation using an iPad. (**b**) Example of manual annotation of nematodes in the digital images (blue color circling). (**c**) Example of digital annotation of nematodes in a synthetic RGB image extracted from the hyperspectral cube (yellow color circling).

To develop a solution for automatic nematode detection using deep learning and hyperspectral imaging, a dataset comprising annotated digital images containing the precise location of the nematodes needed to be generated. Before the manual inspection of the fish samples, they were scanned with the Maritech Eye (Fig. 2). Data was extracted from the hyperspectral images to create RGB digital images of the belly flaps. These images were displayed on an iPad, such they could be annotated to mark the location of nematodes during manual inspection (Fig. 3a). To provide the ground truth needed to train the neural network models, nematodes in the fish samples were identified using the candling method. Here, the belly flaps are inspected on a light table and a skilled examiner identifies the location of the nematodes. In contrast to industrial conditions, where each fillet is inspected in a span of approximately ten seconds, a thorough inspection of a fillet over the span of several minutes was performed to ensure no nematodes were missed. The drawback to this reference method is that it limits detection to visible nematodes. Per the Commission Regular (EC) a visible nematode is defined as “*a parasite or group of parasites which has a dimension, colour or texture which is clearly distinguishable from fish tissue… Visual inspection means non-destructive examination of fish or fishery products with or without optical means of magnifying and under good light conditions for human vision, including, if necessary, candling”*^42^*.* Therefore, nematodes that are buried too deep in the flesh to be visible by the naked eye are not included in the calculations of detection precision and accuracy. Although visual inspection will not identify every nematode present in a fish, it is expected to identify the majority of them in this study, as research has found that 96% of all nematodes in cod are not embedded deeper than 10 mm in the tissue^2^ and the belly flaps were typically less than 10 mm in thickness. Despite reports indicating human mistakes in the visual identification of nematodes during the processing of fillets, the samples chosen for annotation in this research were meticulously examined by an expert, who took additional time in the examination of every sample to ensure accuracy. Furthermore, during the process of digitizing annotations on the digital images, if the operator observed any additional areas of concern within the sample, a secondary evaluation was performed by the expert to determine the presence or absence of nematodes. Therefore, we expect the annotation error rate in our study to be significantly reduced compared to those usually reported for the visual identification of nematodes.

The manually annotated digital images, examples Supplementary Fig. 1, were used to pinpoint the precise location of the nematodes within a fish sample (Fig. 3b), and to serve as a guide for the digital annotation of the nematodes in the hyperspectral images (Fig. 3c). The annotation of the hyperspectral images was performed manually using the Breeze software for hyperspectral image analysis (Prediktera AB, Umeå, Sweden). Often nematodes cannot be identified by visual inspection of RGB or single band images created from hyperspectral images without preprocessing. For this reason, once the approximate locations of the nematodes were determined using the reference digital annotations, the exact location of each nematode in a hyperspectral image was assessed by using different synthetic representations of the hyperspectral data, e.g., using different principal component analysis (PCA) combinations of the hyperspectral data. These alternative representations of the hyperspectral data allowed the nematodes’ location and shape to be highlighted among the surrounding fish muscle. Finally, once the nematode position and shape were identified, they were manually annotated using polygon shapes. This labeling process was labor intensive, both due to the difficulty of finding suitable preprocessing to help distinguish the nematodes from the surrounding fish muscle and the challenge of exhaustively searching each fillet to find every nematode for manual annotation of the digital images.

**Fig. 4:**
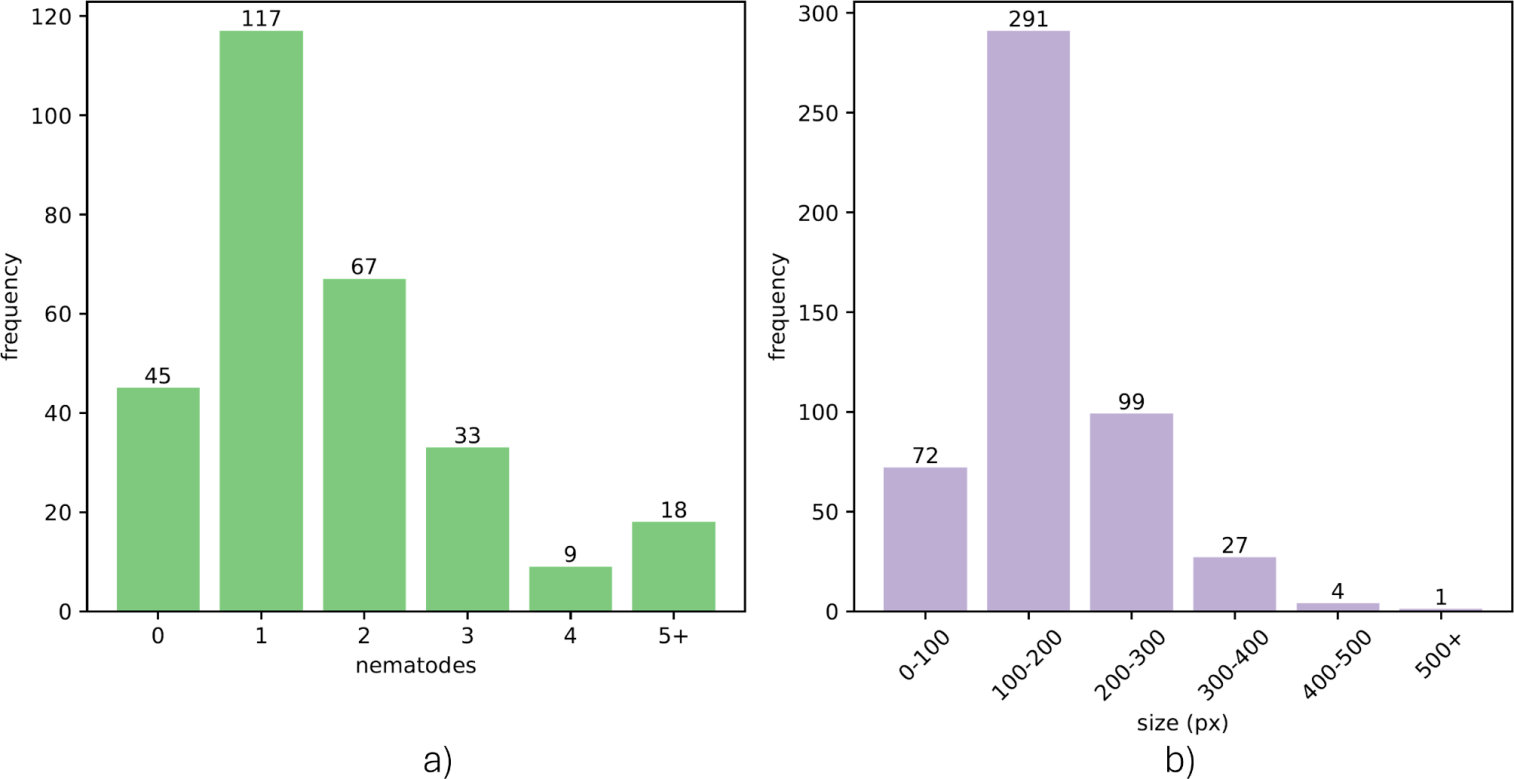
Dataset summary, including the number of nematodes per sample (**a**), and the size of the nematodes (**b**).

Of the 335 images acquired during the trials, only 289 of the images were annotated. The rejection of some of those samples was motivated by the difficulty of locating some of the nematodes in the hyperspectral images, which could produce confounding information for the deep learning model if they were not annotated. In the remaining 289 images, a total of 244 nematodes were identified. The distribution of the number of nematodes per sample and their size is summarized in Fig. 4. A visual example of the location, size, and shape of nematodes present in some of the samples comprising the dataset can be observed in Supplementary Fig. 1.

+

As we are utilizing a supervised machine learning model, an adequate data partition must be performed to ensure the appropriate and unbiased generation and evaluation of the models. In this case, the splitting of samples into training, validation, and test sets was done by random sampling, with the training set containing 196 samples (68%), the validation set 49 samples (17%), and the test set 44 samples (15%).

### Data processing

**Fig. 5:**
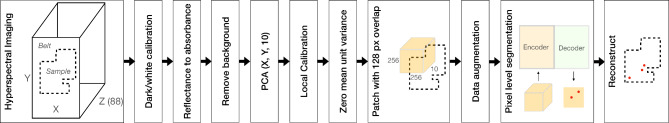
Processing workflow for the detection of nematodes from hyperspectral image data.

In this section, we describe the workflow for developing algorithms for the automatic detection of nematodes from hyperspectral images (Fig. 5). Our study’s image processing workflow employs established techniques tailored for hyperspectral data. Flat field calibration normalizes illumination and sensor discrepancies, while Principal Component Analysis (PCA) reduces data dimensionality, preserving vital variance. Local calibration corrects for sample variability, and zero mean unit variance normalization standardizes data for model training. Finally, the LinkNet deep learning architecture, designed for semantic segmentation, efficiently combines spatial and spectral information to differentiate nematodes from fish tissue. These methods are theoretically grounded in computer vision and machine learning, forming a cohesive workflow for nematode detection in white fish.

### Flat field calibration

The first preprocessing stage applied to the hyperspectral data is a flat field calibration of the hyperspectral images followed by the subsequent transformation of the images into pseudo-absorbance. This calibration makes use of two reference images. The white reference image consists of a hyperspectral image of a PTFE (Teflon) target plate of 25 mm thickness, which is characterized by a flat spectral response in the camera spectral range^43^, while the dark reference image is acquired when light is blocked from entering the camera sensor. The white reference image contains information about the spectral response of the hyperspectral system, including the spectral shape of the light source and the spatial distribution of the light within the field of view of the camera. The dark reference contains the noise recorded by the hyperspectral camera sensor. When applying the flat field calibration (Eq. 1), the environmental conditions of the hyperspectral data are removed. In Eq. 1, ${HC}_{cal}$ is the calibrated hyperspectral image, ${HC}_{raw}$ is the raw hyperspectral data, and WhiteRef and DarkRef are the white and dark references, respectively. Finally, the hyperspectral images are converted into pseudo-absorbance by applying the transformation on Equation 2.

1 $${HC}_{cal}=\frac{{HC}_{raw}-DarkRef}{WhiteRef-DarkRef}$$

2 $${HC}_{absorbance}=-{log}_{10}{(HC}_{cal})$$

### Background removal

The second step in the processing workflow consists of image segmentation where the belly pieces are identified against the background, i.e., the conveyor belt. In this case, we used a segmentation model using a PCA transformation based on the spectral information from the belly samples and the background, followed by thresholding to identify the pixels corresponding to the sample. This segmentation capability is a feature provided by the Breeze software.

### PCA dimensionality reduction

Once the segmentation of the fish sample was performed, a PCA model was constructed to perform a dimensionality reduction of the spectral information. Reducing the dimensionality of hyperspectral images is a widely adopted preprocessing step in the field of hyperspectral image classification. The main purpose of this process is to reduce the hyperspectral data dimensions, while ensuring that the most significant information is retained^44^. PCA has been widely employed as a method for dimensionality reduction in hyperspectral image analysis^45,46^. PCA reduces high-dimensional hyperspectral data to a lower-dimensional space by utilizing orthogonal variables from the data covariance matrix. In this case, we targeted to reduce the dimensionality of the data from the original 88 spectral bands to 10 components. This PCA model was constructed from the pixels (1 × 1 × 88) taken from the samples belonging to the training set (n = 196). As the pixels assigned to the non-nematode class were overrepresented compared to the nematode pixels, the nematode pixels were randomly oversampled to create a class balance array of spectral data. Every pixel in the training set was stacked to create an N × 88 matrix stacking all the spectral information, with N being the number of nematode and non-nematode pixels (several millions). Once this spectral dataset was created, the PCA model was computed, and the hyperspectral data was transformed from an initial dimension of W × H × 88 to a final dimension of W × H × 10.

### Local calibration

No significant differences were found when analyzing the spectral signature of the nematodes and the flesh within the belly samples. As suggested in a previous study^38^, in interactance or transmission measurements, the light interacting with a nematode near the fillet surface is similar to the light measured from an area next to the nematode. For that reason, a local calibration filter is proposed to calibrate the spectrum of pixels with the goal of reducing spectral variations, such as those caused by the fillet color or light scattering effects.

This local calibration has three parameters: ${r}_{1},{r}_{2}$ and ${r}_{3}$. The implementation of the local calibration is based on using 2D image filter kernels, which are applied to the hyperspectral image. The local neighborhood kernel (${K}_{LOCAL}$) has a dimension of ${2r}_{1}+1\times {2r}_{1}+1$, and is defined by Eq. 3 for the spatial coordinates $(x,y)$. The background filter (${K}_{BKG}$) has a dimension of ${2r}_{3}+1\times {2r}_{3}+1$, and is defined by Eq. 4.

3 $${K}_{LOCAL}\left(i,j\right)=\left\{\begin{array}{cc}\frac{1}{\sqrt{{(i-x)}^{2}+{(j-y)}^{2}}} & \forall i,j=0\dots {2r}_{1}\\ 0& \forall \left(i,j\right)=({r}_{1},{r}_{1})\end{array}\right.$$

4 $${K}_{BKG}\left(i,j\right)=\left\{\begin{array}{cc}\frac{1}{\sqrt{{(i-x)}^{2}+{(j-y)}^{2}}} & \forall i,j \ne {r}_{2}-{r}_{1}\dots {r}_{2}+{r}_{1}\\ 0& \forall i,j={r}_{2}-{r}_{1}\dots {r}_{2}+{r}_{1}\end{array}\right.$$

For every spatial pixel $\left(x,y\right)$ in every dimension of the W × H × 10 data cube after the PCA transformation ${HC}_{PCA}$, the local calibration is applied as described in Eq. 5, where ${I}_{LOCAL}$ and ${I}_{BKG}$ are subregions of ${HC}_{PCA}$ centered in the spatial coordinates $\left(x,y\right)$ of size ${2r}_{1}+1\times {2r}_{1}+1$ and ${2r}_{3}+1\times {2r}_{3}+1$:

5 $$LC\left(x,y\right)= \frac{{I}_{LOCAL}*{K}_{LOCAL}}{{I}_{BKG}*{K}_{BKG}}=\frac{\sum_{i=0}^{2{r}_{1}}\sum_{j=0}^{2{r}_{1}}{K}_{LOCAL}\left(i,j\right){I}_{LOCAL}(x-i, y-j)}{\sum_{i=0}^{2{r}_{3}}\sum_{j=0}^{2{r}_{3}}{K}_{BKG}\left(i,j\right){I}_{BKG}(x-i, y-j)}$$

6 $${I}_{LOCAL}={HC}_{PCA}\left(x-{r}_{1}:x+{r}_{1}, y-{r}_{1}:y+{r}_{1}\right)$$

7 $${I}_{BKG}={HC}_{PCA}(x-{r}_{3}:x+{r}_{3}, y-{r}_{3}:y+{r}_{3})$$

In this work, the values for ${r}_{1}, {r}_{2},$ and ${r}_{3}$ are 1, 2 and 5 respectively.

### Zero mean unit variance

The data was normalized to contain a mean of zero and a standard deviation of 1, known as zero mean unit variance. The mean ($\overline{X }$) and standard deviation (${\sigma }_{X}$) value are obtained from the training data only. The formula is given by Eq. 7, where X represents each individual value in the x, y, lambda dimensions.

7 $$\frac{X- \overline{X}}{{\sigma }_{X}}$$

### Patches generation

For each sample image (W ´ H ´ 10), patches of dimensions 256 ´256 ´ 10 were created with a stride of 128 to be the input to the deep learning model. Since the dimensions of the sample were typically not divisible by 256, each sample image was padded to a width or height that could be divisible by 256 to create equal size patches. Padding was performed on all borders. With a stride smaller than the patch size, overlapping patches were created.

For each 256 ´ 256 ´ 10 image in the training data, data augmentation was performed through 90 degrees rotation, as well as flipping each patch on the vertical axis. This resulted in 8 variations of the same patch, including the original patch. The model was trained using 25,328 patches (after data augmentation), and the number of patches for validation and test were 795 and 706, respectively. The number of patches containing nematodes was 8064, 290, and 189 for training, validation, and test, which constitutes a highly unbalanced dataset.

**Fig. 6:**
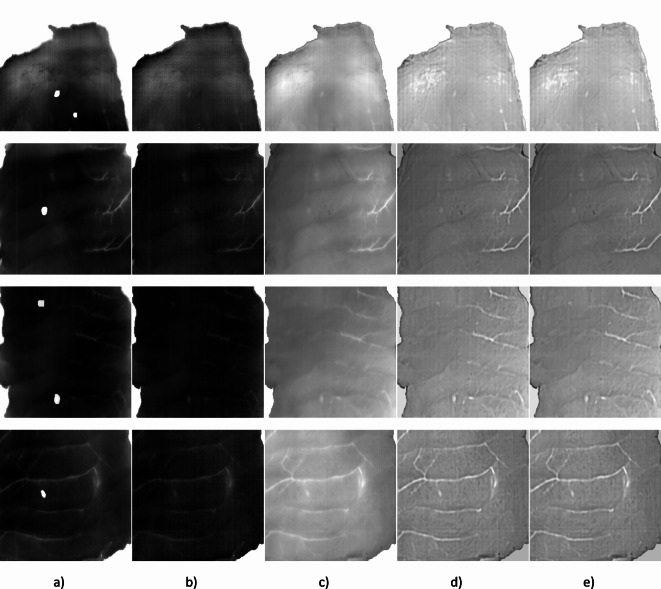
Patches of 256 × 256 pixels used for training and evaluating the deep neural network model for the different preprocessing stages: (**a**) Single band (545 nm) nematode annotations, (**b**) Single band image (545 nm), (**c**) Second principal component image after PCA, (**d**) Second principal component image after applying local calibration, (**e**) Zero mean unit variance image.

Figure 6 shows examples of patches used to train the deep neural network for the different preprocessing stages. Figure 6a shows a single band image from the hyperspectral cube (545 nm), where the location of the nematodes, according to the manual annotations, is highlighted. Figure 6a illustrates the heterogeneity in shape, size, and location of the nematodes within the fish samples. Figure 6b shows the same single-band image where the nematodes are not highlighted. Figure 6c shows the second principal component image after applying PCA. The second PCA component was selected for the representation because it highlights the nematodes over the fish tissue. Figure 6d shows the result of the local calibration application to the image. In Fig. 6d, it is observed that the visualization of both different tissue structures (connective tissue, blood vessels, etc.) and nematodes are enhanced. Finally, Fig. 6e shows the results of the zero mean unit variance normalization.

### Deep neural network architecture

The objective of image segmentation is to categorize every pixel within an image into distinct object classes. In recent years, various deep learning-based approaches have been proposed for performing image segmentation^47^.Our solution involves a pixel wise segmentation model based on the LinkNet architecture^48^. The LinkNet architecture, combined with a DenseNet encoder, is effective in semantic segmentation of hyperspectral data by efficiently leveraging dense feature reuse for spectral depth and LinkNet’s shortcuts for spatial detail, creating a computationally effective model for complex, high-dimensional datasets. The LinkNet architecture has an encoder decoder structure and is especially designed to reduce training and inference time, as well as to be memory efficient. The encoder part encodes the spatial and spectral information into a feature space, whereas the decoder part translates this back to a spatial representation to enable segmentation.

**Fig. 7:**
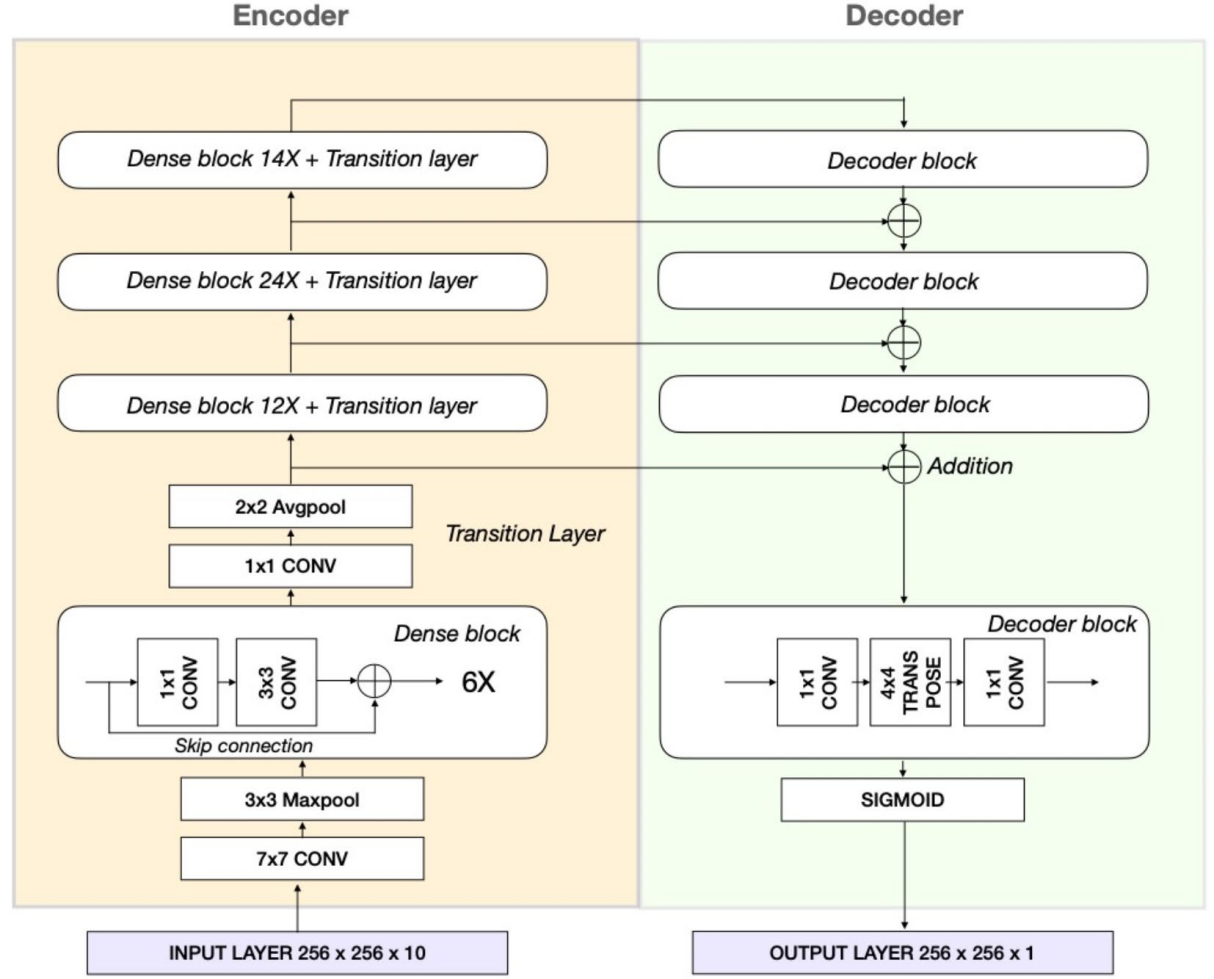
LinkNet encoder-decoder architecture with Densenet-121 encoder.

The encoder consists of a Densenet 121-layer convolutional neural network^49^. Each dense block (Fig. 7) consists of several convolutional operators in which skip connections occur. Denseblocks contain batch normalization and RELU activation functions, which are omitted in Fig. 7. Each encoder layer is connected to the decoder part of the architecture to recover the lost spatial information, which occurs in the encoder part due to downsampling with pooling layers. Within the decoder part of the network, the features are upsampled through transpose convolutions which contain trainable parameters, compared to other upsampling approaches.

The LinkNet architecture with Densenet121 encoder structure is trained with a learning rate of 5 × 10–4 and ADAM optimizer, batch size of 16, for a total of 500 epochs with early stopping set when the validation loss did not decrease for 50 epochs.

For segmentation problems, loss functions based on the overlap measurements have demonstrated their robustness, especially when there is a class imbalance^50^. For this reason, we used the DICE loss with a beta of 2, which can be defined as:

8 $$L\_DICE(tp,fp,fn)=\frac{\left(1+{\beta }^{2}\right)\cdot tp}{\left(1+{\beta }^{2}\right)\cdot fp+{\beta }^{2}\cdot fn+fp}$$

With tp = true positives, fp = false positives, and fn = false negatives. DICE is equivalent to the F1 score and can thus be written as:

9 $$L\_DICE(\text{ precision, recall })=1-\left(1+{\beta }^{2}\right)\frac{\text{ precision }\cdot \text{ recall }}{{\beta }^{2}\cdot \text{ precision }+\text{ recall}}$$

The DICE is a similarity metric that measures the overlap between the predictions made by the neural network model and the ground truth. The $\beta$ value determines how important is recall in comparison with precision, which means that the DICE loss function will penalize the false negatives in the learning process.

This work was developed in Python. The Scikit-learn library^51^ was used for the implementation of PCA, the performance metrics, and the data partitions; the OpenCV library was used for the local calibration^51^ and the TensorFlow implementation of the Keras Deep Learning API^52,53^ together with the Segmentation Models API^54^ were used for the Deep Neural Network implementation.

### Evaluation metrics

In this section, we describe the metrics used for the evaluation of the performance of the proposed workflow. To determine whether a nematode was considered detected or not, we used the Intersection of the Union (IOU, also known as Jaccard Index) between the predicted bounding box by the deep learning model (${B}_{p}$) and the ground truth bounding box (${B}_{gt}$) according to the manual labels. The IOU measures the overlap between the predicted and the labeled bounding box.

$$J\left({B}_{p},{B}_{gt}\right)=\text{IOU}=\frac{\text{area}\left({B}_{p}\cap {B}_{gt}\right)}{\text{area}\left({B}_{p}\cup {B}_{gt}\right)}$$

The output of the deep learning network provides for each pixel a value between 0 and 1, indicating the probability that it belongs to the class nematode. For the predictions to be binary for each pixel and to make the final class assignment (nematode or not), a threshold for the deep learning network output is defined ${(Th}_{model}).$ The model threshold determines the bounding boxes of the predictions, and enables the calculation of the IOU. Additionally, it is necessary to establish a second threshold to decide how much overlap between the two bounding boxes (predicted and ground truth) is required to determine if the segmentation of the nematode is considered correct ${(Th}_{IOU}).$

For each pair of ${Th}_{IOU}$ and ${Th}_{model}$ values, the predictions can be quantified in terms of true positives (TP), false positives (FP), and false negatives (FN). In order to evaluate the overall detection performance of nematodes in the dataset, we report precision, recall, and F1 score for different combinations of ${Th}_{IOU}$ and ${Th}_{model}$. On the one hand, precision $(P)$ is the ratio between the actual number of successes in detecting nematodes and the total number of nematodes flagged by the model. High precision indicates an accurate detection of positive cases with a low number of false positives. On the other hand, recall $\left(R\right)$ is the ratio between the total number of successes in detecting nematodes and the total number of nematodes in the dataset. A high recall implies a high detection rate of the nematodes in the dataset and indicates the ability of the model to avoid false negatives. Finally, the F1 score is the harmonic mean of precision and recall and measures the overall quality of the predictions. Contrary to the overall accuracy, precision, recall, and F1 are not sensitive to unbalanced datasets.

$$P = \frac{{{\text{TP}}}}{{{\text{TP}} + {\text{FP}}}} = \frac{{{\text{TP}}}}{{\text{ all detections }}}$$

$$R = \frac{{{\text{TP}}}}{{{\text{TP}} + {\text{FN}}}} = \frac{{{\text{TP}}}}{{\text{ all ground truths }}}$$

$${\text{F}}1 = 2\frac{{{\text{P}} \cdot {\text{R}}}}{{{\text{P}} + {\text{R}}}} = { }\frac{{2{\text{ TP}}}}{{2{\text{TP}} + {\text{FP}} + {\text{FN}}}}$$

### Evaluation of the model performance

The learning curves corresponding to the training of the LinkNet model with Densenet-121 encoder are shown in Supplementary Fig. 2 and refer to the model performance at the patch level.

With the goal of obtaining a model with high recall, we used the DICE loss function with $\beta =2$, which forces the recall to be twice as important as precision during the learning process. The best model was selected with regards to the minimum DICE loss for the validation data, and with an early stopping criterion. In this case, the optimum model was found after 106 epochs. Supplementary Fig. 2b shows the evolution of the IOU during training. For the optimal model, the IOU is 0.67 for the validation set, which means that the average overlap between the predictions and the ground truth data is 67%. The IOU helps penalize both under and over-segmentation. In this specific application, a perfect overlap between the predictions and the ground truth may not be ideal due to possible pixel-level labeling errors during the manual annotations of the samples. The similarity between the IOU score in the training, validation, and test set indicates no overfitting.

Supplementary Fig. 3 shows a report of the precision, recall, and F1-score performance for different model and IOU thresholds for the training, validation, and test sets. There are no relevant variations in the evaluation metrics when the model threshold changes for a fixed IOU threshold. Additionally, it is worth mentioning that the model produces accurate predictions when the model threshold is higher than 0.1, i.e., the probability of a pixel belonging to the nematode class is higher than 10%. The IOU threshold has a higher impact on the evaluation metrics than the model threshold. As mentioned before, the choice of IOU threshold strongly impacts the evaluation metrics. Precision is not as sensitive to the IOU threshold because false positives are not affected by the IOU threshold. Also, the changes in the IOU threshold are only related to a decrease in true positives associated with a high IOU threshold. In contrast, recall is more sensitive to the IOU threshold because it affects both the true positives and the false negatives.

Regarding the detection performance of the proposed approach, for an IOU threshold of 0.5, the results of the test set show predictions with high precision (0.89) and a good recall (0.73). This means that the current model can identify 73% of the nematodes present in the samples with a low number of false positives. From the perspective of applying this model in a real-world scenario, high precision means that only a small fraction of the fish samples without the nematodes will be manually reinspected. This will in many situations greatly reduce the workload on trimmers. However, the main feature of an industrial nematode detection solution would be to identify most of the nematodes (high recall) to identify which fish pieces should be manually inspected and trimmed and ensure that no fish with nematodes are sent to the market.

Although our solution has a high nematode detection rate, several optimization strategies can be employed to increase the detection even further. For example, it may involve expanding data diversity with advanced augmentation, integrating anomaly detection, and adopting newer neural network architectures. Optimizing hyperparameters, employing higher-resolution imaging, and establishing a feedback loop for continuous model refinement could further improve accuracy and adaptability to new data patterns.

### Model development challenges

The dataset imposed some challenges that limit the neural network model’s performance. First, although the initial dataset was composed of 335 images, the digital annotation of the samples was possible in only 289. This means that about 14% of the samples were not used and reveals the difficulty providing ground truth identification of nematodes. The motivation for rejecting those samples was to limit the analysis, especially during the training phase, to data with highly reliable labels. The limited size of the dataset restricted the feasibility of performing multiple training-validation splits, which may affect the generalizability of the results. Future work with larger datasets could explore this aspect more thoroughly. Second, the dataset is highly imbalanced. The patches used to train the model were 25,328 (after data augmentation), from which only 8064 contained nematodes. This means that the number of patches containing nematodes in the training set was only 32%. Additionally, the size of the nematodes is relatively small (according to Fig. 4, up to 500 pixels) compared to the size of the patches (256´256). The imbalanced number of samples available to train the model and the heterogeneity of the nematodes regarding their location in different types of tissue within the fish flesh, and their shape and size impose significant challenges for teaching a neural network model able to provide generalization among those different scenarios. However, despite these challenges, the neural network model was able to provide competent results, which may be further improved with an increased dataset.

## Electronic supplementary material

Below is the link to the electronic supplementary material.

Supplementary Material 1

## Data Availability

Data used in this study are available upon reasonable request by contacting the corresponding author. Code used in this study is available upon reasonable by contacting the corresponding author.

## Code availability

Code used in this study is available upon reasonable request by contacting the corresponding author.
