# Supplementary material for: Hyperspectral imaging and deep learning for parasite detection in white fish under industrial conditions
Source: Sci Rep. 2024 Nov 9;14:27426. doi: 10.1038/s41598-024-76808-w (PMC11550473; doi:10.1038/s41598-024-76808-w)
Supplement: Supplementary file 1 — Supplementary Material 1 [file 41598_2024_76808_MOESM1_ESM.docx]

**
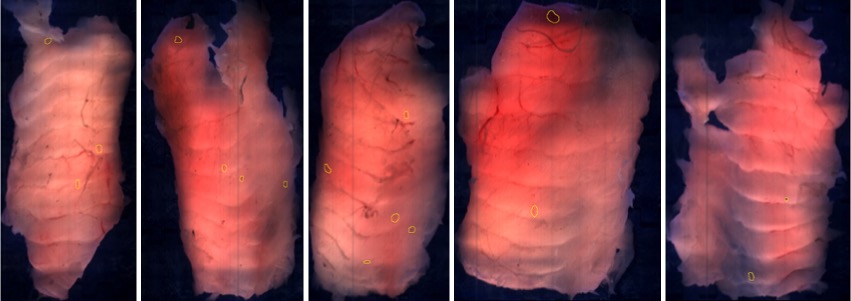
**

**Supplementary Figure 1:** Examples of the distribution, shape, and size of nematodes (yellow color annotations) in different belly pieces.

|  | |
| --- | --- |
| a) | b) |

**Supplementary Figure 2:** Learning curves for the LinkNet model with Densenet-121

**Supplementary Figure 3:** Precision, recall, and F1 score depending on the model threshold and the IOU threshold.
